# (2*S*)-3-Carbamoyl-2-(4-meth­oxy­benzene­sulfonamido)­propanoic acid

**DOI:** 10.1107/S160053681003607X

**Published:** 2010-09-18

**Authors:** Hafiz Mubashar-ur-Rehman, Islam Ullah Khan, Muhammad Nadeem Arshad, K. Travis Holman

**Affiliations:** a Materials Chemistry Laboratory, Department of Chemistry, GC University, Lahore 54000, Pakistan; b Department of Chemistry, Georgetown University, 37th and O St NW, Washington, DC 20057, USA

## Abstract

In the title compound, C_11_H_14_N_2_O_6_S, an amino acid-derived sulfonamide, the acetamido group and the carb­oxy­lic group are oriented at dihedral angles of 45.84 (5)° and 47.97 (5)° respectively, with respect to the aromatic ring. In the crystal, the mol­ecules are connected by N—H⋯O and O—H⋯O hydrogen bonds and weak C—H⋯O inter­actions, forming a three-dimensional network.

## Related literature

For related structures, see: Arshad *et al.* (2009*a* ▶,*b* ▶), Khan *et al.* (2009 ▶). Amino acid-derived sulfonamide derivatives have been used as potent inhibitors of Procollagen C-Proteinase, see: Dankwardt *et al.* (2002 ▶).
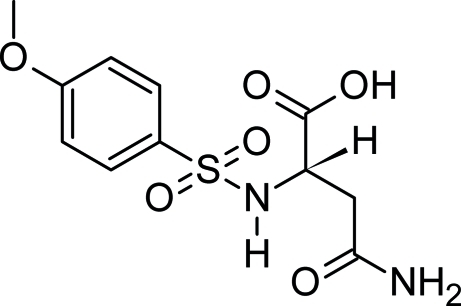


## Experimental

### 

#### Crystal data

C_11_H_14_N_2_O_6_S
*M* *_r_* = 302.30
Monoclinic, 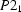

*a* = 7.1462 (1) Å
*b* = 8.9874 (2) Å
*c* = 11.1418 (2) Å
β = 108.090 (1)°
*V* = 680.22 (2) Å^3^
*Z* = 2
Mo *K*α radiation
μ = 0.27 mm^−1^
*T* = 100 K
0.42 × 0.26 × 0.23 mm

#### Data collection

Siemens SMART diffractometer equipped with a Bruker APEXII detector
Absorption correction: multi-scan (*SADABS*; Bruker, 2007 ▶) *T*_min_ = 0.897, *T*_max_ = 0.942
15356 measured reflections
3434 independent reflections
3335 reflections with *I* > 2σ(*I*)
*R*_int_ = 0.028

#### Refinement

*R*[*F* ^2^ > 2σ(*F* ^2^)] = 0.024
*wR*(*F* ^2^) = 0.063
*S* = 1.04
3434 reflections
194 parameters
1 restraint
H atoms treated by a mixture of independent and constrained refinement
Δρ_max_ = 0.30 e Å^−3^
Δρ_min_ = −0.23 e Å^−3^
Absolute structure: Flack (1983 ▶), 1581 Friedel pairs
Flack parameter: −0.01 (4)

### 

Data collection: *APEX2* (Bruker, 2007 ▶); cell refinement: *SAINT* (Bruker, 2007 ▶); data reduction: *SAINT*; program(s) used to solve structure: *SHELXS97* (Sheldrick, 2008 ▶); program(s) used to refine structure: *SHELXL97* (Sheldrick, 2008 ▶); molecular graphics: *ORTEP-3* (Farrugia, 1997 ▶), *PLATON* (Spek, 2009 ▶) and *X-SEED* (Barbour, 2001 ▶); software used to prepare material for publication: *WinGX* (Farrugia, 1999 ▶) and *PLATON*.

## Supplementary Material

Crystal structure: contains datablocks I, global. DOI: 10.1107/S160053681003607X/hb5624sup1.cif

Structure factors: contains datablocks I. DOI: 10.1107/S160053681003607X/hb5624Isup2.hkl

Additional supplementary materials:  crystallographic information; 3D view; checkCIF report

## Figures and Tables

**Table 1:** Hydrogen-bond geometry (Å, °)

*D*—H⋯*A*	*D*—H	H⋯*A*	*D*⋯*A*	*D*—H⋯*A*
N2—H1*N*2⋯O3^i^	0.812 (19)	2.147 (19)	2.9430 (15)	166.9 (16)
N2—H2*N*2⋯O2^ii^	0.89 (2)	2.02 (2)	2.8808 (16)	162.9 (17)
C11—H11*B*⋯O3^iii^	0.98	2.48	3.3701 (17)	150
N1—H1*N*⋯O5^ii^	0.927 (18)	1.907 (18)	2.8196 (14)	167.8 (16)
O4—H4*O*⋯O5^iv^	0.81 (2)	1.79 (2)	2.5875 (14)	169 (2)
C9—H9*A*⋯O2^ii^	0.99	2.54	3.4055 (16)	145

Symmetry codes: (i) 
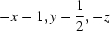
; (ii) 
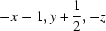
; (iii) 
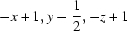
; (iv) 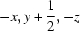.

**Fig. 1.**
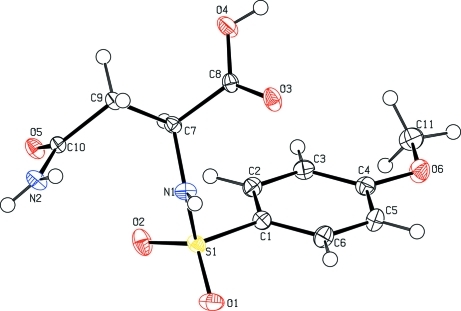
The molecular structure of (I) with displacement ellipsoids drawn at the 50% probability level.

**Fig. 2.**
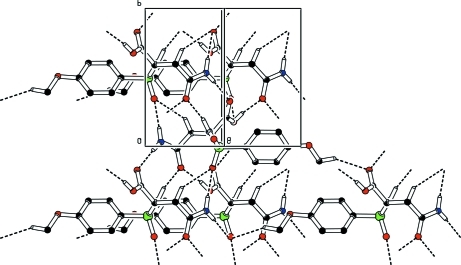
Unit cell packing for (I) with hydrogen bonding shown as dashed lines and the hydrogen atoms not involved in hydrogen bonding have been omitted.

## supplementary crystallographic information

### Comment

Amino acid derived sulfonamide derivatives have been used as potent inhibitors of Procollagen C-Proteinase (Dankwardt *et al.*, 2002). This structure is in countinuation to already reported crystal structures of sulfonamides derived from amino acids (Arshad *et al.*, 2009*a*), (Arshad *et al.*, 2009*b*) (Khan *et al.*, 2009) by our group.

The dihedral angle between the acetamido group attached at the C7 and the carboxylic  group C7/C8/O3/O4 is 38.64 (0.05)° while these two groups are oriented at dihedral angle of 45.84 (0.05)° and 47.97 (0.05)° respectively with respect to the aromatic ring. The symmetry related intermolecular N—H···O, O—H···O and weak C—H···O type interactions stabilized the structure by the formation of three dimentional network (Fig. 2, Table, 1).

### Experimental

To the solution of L-asparagine (0.5 g, 3.78 mmol) in distilled water (10 ml), 4-methoxybenzenesulfonyl chloride(0.78 g, 3.78 mmol) was suspended. The reaction mixture was allowed to stirr at room temperature for 2 hrs. The pH of the solution was maintained at 8–9 by 1*M* sodium carbonate solution through out the reaction. After completion of the reaction which was observed by the consumption of suspended 4-methoxybenzenesulfonyl chloride, 1 *N* HCl solution was used to adjusted the pH about 2–3, which results in the formation of a white precipitate, which was filtered off, dried and recrystallized in methanol by slow evaporation to yield colorless needles of (I).

### Refinement

The C-H H-atoms were positioned geometricaly with C—H = 0.95 Å, C—H = 0.99Å and C—H = 1.00 Å for aromatic, methylene and chiral carbon atoms respectively, and were refined using a riding model with *U*_iso_(H) = 1.2 *U*_eq_(C). Similarly the C-H H-atoms were positioned geometricaly with C—H = 0.98 Å for methyl group and were refined using a riding model with *U*_iso_(H) = 1.5 *U*_eq_(C). The N-H and O–H H atoms were located in difference map with N–H= 0.81 (2)—0.93 (2)Å and O—H= 0.81 (2) with *U*_iso_(H) = 1.2 for N atoms and *U*_iso_(H) = 1.5 for O atoms. The three reflections (001), (002) and (003) were omitted during the final refinement as these were obscured by the beam stop.

### Crystal data

**Table:**

C_11_H_14_N_2_O_6_S	*F*(000) = 316
*M**_r_* = 302.30	*D*_x_ = 1.476 Mg m^−^^3^
Monoclinic, *P*2_1_	Mo *K*α radiation, λ = 0.71073 Å
Hall symbol: P 2yb	Cell parameters from 8744 reflections
*a* = 7.1462 (1) Å	θ = 3.0–28.6°
*b* = 8.9874 (2) Å	µ = 0.27 mm^−^^1^
*c* = 11.1418 (2) Å	*T* = 100 K
β = 108.090 (1)°	Needle, colorless
*V* = 680.22 (2) Å^3^	0.42 × 0.26 × 0.23 mm
*Z* = 2	

### Data collection

**Table:**

Siemens SMART diffractometer equipped with a Bruker APEXII detector	3434 independent reflections
Radiation source: fine-focus sealed tube	3335 reflections with *I* > 2σ(*I*)
graphite	*R*_int_ = 0.028
φ and ω scans	θ_max_ = 28.6°, θ_min_ = 3.0°
Absorption correction: multi-scan (*SADABS*; Bruker, 2007)	*h* = −9→9
*T*_min_ = 0.897, *T*_max_ = 0.942	*k* = −11→12
15356 measured reflections	*l* = −14→14

### Refinement

**Table:**

Refinement on *F*^2^	Secondary atom site location: difference Fourier map
Least-squares matrix: full	Hydrogen site location: inferred from neighbouring sites
*R*[*F*^2^ > 2σ(*F*^2^)] = 0.024	H atoms treated by a mixture of independent and constrained refinement
*wR*(*F*^2^) = 0.063	*w* = 1/[σ^2^(*F*_o_^2^) + (0.0368*P*)^2^ + 0.1181*P*] where *P* = (*F*_o_^2^ + 2*F*_c_^2^)/3
*S* = 1.04	(Δ/σ)_max_ < 0.001
3434 reflections	Δρ_max_ = 0.30 e Å^−^^3^
194 parameters	Δρ_min_ = −0.23 e Å^−^^3^
1 restraint	Absolute structure: Flack (1983), 1581 Friedel pairs
Primary atom site location: structure-invariant direct methods	Flack parameter: −0.01 (4)

### Special details

**Table:**

Geometry. All esds (except the esd in the dihedral angle between two l.s. planes) are estimated using the full covariance matrix. The cell esds are taken into account individually in the estimation of esds in distances, angles and torsion angles; correlations between esds in cell parameters are only used when they are defined by crystal symmetry. An approximate (isotropic) treatment of cell esds is used for estimating esds involving l.s. planes.
Refinement. Refinement of F^2^ against ALL reflections. The weighted R-factor wR and goodness of fit S are based on F^2^, conventional R-factors R are based on F, with F set to zero for negative F^2^. The threshold expression of F^2^ > 2sigma(F^2^) is used only for calculating R-factors(gt) etc. and is not relevant to the choice of reflections for refinement. R-factors based on F^2^ are statistically about twice as large as those based on F, and R- factors based on ALL data will be even larger.

### Fractional atomic coordinates and isotropic or equivalent isotropic displacement parameters (Å^2^)

**Table:**

	*x*	*y*	*z*	*U*_iso_*/*U*_eq_	
S1	−0.29092 (4)	−0.00600 (3)	0.24767 (3)	0.01463 (7)	
O1	−0.42282 (14)	0.05232 (12)	0.31015 (9)	0.0222 (2)	
N1	−0.30244 (16)	0.10725 (12)	0.13389 (10)	0.0151 (2)	
C1	−0.04937 (16)	−0.00385 (17)	0.35229 (10)	0.0149 (2)	
H1N	−0.375 (3)	0.193 (2)	0.1302 (16)	0.018*	
H1N2	−0.739 (3)	−0.027 (2)	−0.1489 (16)	0.018*	
O2	−0.32080 (14)	−0.15497 (11)	0.19714 (9)	0.0228 (2)	
N2	−0.65042 (16)	0.03205 (13)	−0.13841 (11)	0.0173 (2)	
C2	0.08556 (19)	−0.11149 (14)	0.34294 (12)	0.0168 (2)	
H2	0.0478	−0.1847	0.2784	0.020*	
H2N2	−0.669 (2)	0.130 (2)	−0.1430 (17)	0.020*	
O3	−0.03178 (13)	0.32670 (11)	0.13252 (9)	0.0204 (2)	
C3	0.27541 (19)	−0.11253 (15)	0.42761 (13)	0.0181 (2)	
H3	0.3675	−0.1859	0.4212	0.022*	
O4	0.12194 (15)	0.17646 (12)	0.03322 (11)	0.0241 (2)	
C4	0.32887 (17)	−0.00447 (18)	0.52207 (11)	0.0173 (2)	
H4O	0.208 (3)	0.238 (2)	0.0560 (19)	0.026*	
O5	−0.42990 (13)	−0.15430 (10)	−0.10103 (9)	0.01674 (18)	
C5	0.1919 (2)	0.10294 (16)	0.53159 (12)	0.0196 (3)	
H5	0.2285	0.1754	0.5968	0.024*	
O6	0.51004 (13)	0.00661 (13)	0.60857 (8)	0.0223 (2)	
C6	0.0043 (2)	0.10393 (16)	0.44689 (12)	0.0189 (2)	
H6	−0.0877	0.1776	0.4529	0.023*	
C7	−0.17978 (17)	0.09007 (14)	0.05256 (11)	0.0132 (2)	
H7	−0.1132	−0.0091	0.0698	0.016*	
C8	−0.02147 (17)	0.21146 (14)	0.07875 (12)	0.0139 (2)	
C9	−0.30540 (17)	0.09626 (14)	−0.08778 (11)	0.0138 (2)	
H9A	−0.3628	0.1968	−0.1088	0.017*	
H9B	−0.2219	0.0752	−0.1420	0.017*	
C10	−0.46823 (16)	−0.01830 (16)	−0.11135 (10)	0.0134 (2)	
C11	0.6510 (2)	−0.10641 (19)	0.60538 (14)	0.0249 (3)	
H11A	0.5988	−0.2044	0.6167	0.037*	
H11B	0.7742	−0.0886	0.6734	0.037*	
H11C	0.6762	−0.1032	0.5238	0.037*	

### Atomic displacement parameters (Å^2^)

**Table:**

	*U*^11^	*U*^22^	*U*^33^	*U*^12^	*U*^13^	*U*^23^
S1	0.01351 (12)	0.01393 (13)	0.01602 (13)	−0.00161 (11)	0.00396 (9)	0.00279 (11)
O1	0.0180 (4)	0.0296 (5)	0.0215 (4)	0.0010 (4)	0.0097 (4)	0.0059 (4)
N1	0.0158 (5)	0.0138 (5)	0.0171 (5)	0.0037 (4)	0.0074 (4)	0.0040 (4)
C1	0.0147 (5)	0.0149 (5)	0.0141 (5)	0.0006 (5)	0.0030 (4)	0.0027 (5)
O2	0.0230 (5)	0.0141 (5)	0.0264 (5)	−0.0041 (4)	0.0005 (4)	0.0023 (4)
N2	0.0125 (5)	0.0143 (6)	0.0252 (6)	−0.0011 (4)	0.0061 (4)	0.0012 (4)
C2	0.0199 (6)	0.0143 (6)	0.0167 (6)	−0.0004 (5)	0.0062 (5)	−0.0001 (5)
O3	0.0141 (4)	0.0163 (5)	0.0297 (5)	−0.0005 (3)	0.0053 (4)	−0.0082 (4)
C3	0.0180 (6)	0.0181 (6)	0.0188 (6)	0.0021 (5)	0.0066 (5)	0.0012 (5)
O4	0.0170 (5)	0.0220 (5)	0.0373 (6)	−0.0072 (4)	0.0144 (4)	−0.0125 (4)
C4	0.0161 (5)	0.0203 (6)	0.0149 (5)	0.0006 (5)	0.0040 (4)	0.0033 (6)
O5	0.0131 (4)	0.0122 (4)	0.0244 (4)	−0.0006 (3)	0.0051 (3)	−0.0009 (4)
C5	0.0214 (6)	0.0199 (6)	0.0158 (6)	0.0006 (5)	0.0033 (5)	−0.0032 (5)
O6	0.0168 (4)	0.0290 (5)	0.0180 (4)	0.0028 (4)	0.0009 (3)	−0.0013 (4)
C6	0.0211 (6)	0.0171 (6)	0.0179 (6)	0.0029 (5)	0.0054 (5)	−0.0013 (5)
C7	0.0118 (5)	0.0121 (5)	0.0159 (5)	0.0001 (4)	0.0047 (4)	−0.0003 (4)
C8	0.0108 (5)	0.0143 (6)	0.0148 (5)	0.0006 (4)	0.0012 (4)	−0.0002 (4)
C9	0.0121 (5)	0.0131 (5)	0.0157 (5)	−0.0017 (4)	0.0037 (4)	−0.0002 (5)
C10	0.0135 (5)	0.0139 (6)	0.0128 (5)	−0.0029 (4)	0.0042 (4)	−0.0009 (4)
C11	0.0170 (6)	0.0362 (8)	0.0200 (6)	0.0060 (5)	0.0034 (5)	0.0027 (6)

### Geometric parameters (Å, °)

**Table:**

S1—O1	1.4336 (10)	O4—H4O	0.81 (2)
S1—O2	1.4424 (11)	C4—O6	1.3565 (14)
S1—N1	1.6078 (11)	C4—C5	1.402 (2)
S1—C1	1.7576 (11)	O5—C10	1.2500 (17)
N1—C7	1.4514 (15)	C5—C6	1.3790 (18)
N1—H1N	0.927 (18)	C5—H5	0.9500
C1—C2	1.3924 (18)	O6—C11	1.4389 (18)
C1—C6	1.3948 (18)	C6—H6	0.9500
N2—C10	1.3217 (16)	C7—C8	1.5330 (17)
N2—H1N2	0.812 (19)	C7—C9	1.5435 (16)
N2—H2N2	0.89 (2)	C7—H7	1.0000
C2—C3	1.3914 (18)	C9—C10	1.5144 (17)
C2—H2	0.9500	C9—H9A	0.9900
O3—C8	1.2101 (16)	C9—H9B	0.9900
C3—C4	1.3954 (19)	C11—H11A	0.9800
C3—H3	0.9500	C11—H11B	0.9800
O4—C8	1.3151 (16)	C11—H11C	0.9800
			
O1—S1—O2	119.27 (6)	C4—O6—C11	116.86 (11)
O1—S1—N1	105.72 (6)	C5—C6—C1	119.57 (12)
O2—S1—N1	108.34 (6)	C5—C6—H6	120.2
O1—S1—C1	109.41 (6)	C1—C6—H6	120.2
O2—S1—C1	105.36 (6)	N1—C7—C8	111.01 (10)
N1—S1—C1	108.38 (6)	N1—C7—C9	110.77 (10)
C7—N1—S1	122.14 (9)	C8—C7—C9	109.26 (10)
C7—N1—H1N	120.1 (11)	N1—C7—H7	108.6
S1—N1—H1N	117.0 (11)	C8—C7—H7	108.6
C2—C1—C6	120.31 (11)	C9—C7—H7	108.6
C2—C1—S1	120.20 (10)	O3—C8—O4	124.77 (12)
C6—C1—S1	119.46 (10)	O3—C8—C7	123.27 (11)
C10—N2—H1N2	118.7 (12)	O4—C8—C7	111.95 (10)
C10—N2—H2N2	118.0 (11)	C10—C9—C7	108.97 (10)
H1N2—N2—H2N2	123.3 (16)	C10—C9—H9A	109.9
C3—C2—C1	120.40 (12)	C7—C9—H9A	109.9
C3—C2—H2	119.8	C10—C9—H9B	109.9
C1—C2—H2	119.8	C7—C9—H9B	109.9
C2—C3—C4	119.19 (12)	H9A—C9—H9B	108.3
C2—C3—H3	120.4	O5—C10—N2	121.87 (12)
C4—C3—H3	120.4	O5—C10—C9	120.94 (10)
C8—O4—H4O	109.0 (14)	N2—C10—C9	117.14 (12)
O6—C4—C3	124.36 (12)	O6—C11—H11A	109.5
O6—C4—C5	115.50 (12)	O6—C11—H11B	109.5
C3—C4—C5	120.14 (11)	H11A—C11—H11B	109.5
C6—C5—C4	120.38 (12)	O6—C11—H11C	109.5
C6—C5—H5	119.8	H11A—C11—H11C	109.5
C4—C5—H5	119.8	H11B—C11—H11C	109.5
			
O1—S1—N1—C7	176.40 (10)	C3—C4—O6—C11	−3.51 (19)
O2—S1—N1—C7	−54.66 (11)	C5—C4—O6—C11	176.95 (12)
C1—S1—N1—C7	59.18 (11)	C4—C5—C6—C1	0.8 (2)
O1—S1—C1—C2	148.92 (10)	C2—C1—C6—C5	−0.26 (19)
O2—S1—C1—C2	19.54 (12)	S1—C1—C6—C5	177.93 (11)
N1—S1—C1—C2	−96.25 (11)	S1—N1—C7—C8	−108.87 (11)
O1—S1—C1—C6	−29.28 (12)	S1—N1—C7—C9	129.56 (10)
O2—S1—C1—C6	−158.65 (10)	N1—C7—C8—O3	−18.96 (16)
N1—S1—C1—C6	85.55 (11)	C9—C7—C8—O3	103.49 (14)
C6—C1—C2—C3	−0.15 (19)	N1—C7—C8—O4	162.62 (11)
S1—C1—C2—C3	−178.33 (10)	C9—C7—C8—O4	−74.93 (13)
C1—C2—C3—C4	0.06 (19)	N1—C7—C9—C10	−54.36 (13)
C2—C3—C4—O6	−179.08 (12)	C8—C7—C9—C10	−176.96 (10)
C2—C3—C4—C5	0.4 (2)	C7—C9—C10—O5	−65.42 (14)
O6—C4—C5—C6	178.71 (12)	C7—C9—C10—N2	112.13 (12)
C3—C4—C5—C6	−0.8 (2)		

### Hydrogen-bond geometry (Å, °)

**Table:**

*D*—H···*A*	*D*—H	H···*A*	*D*···*A*	*D*—H···*A*
N2—H1N2···O3^i^	0.812 (19)	2.147 (19)	2.9430 (15)	166.9 (16)
N2—H2N2···O2^ii^	0.89 (2)	2.02 (2)	2.8808 (16)	162.9 (17)
C11—H11B···O3^iii^	0.98	2.48	3.3701 (17)	150
N1—H1N···O5^ii^	0.927 (18)	1.907 (18)	2.8196 (14)	167.8 (16)
O4—H4O···O5^iv^	0.81 (2)	1.79 (2)	2.5875 (14)	169 (2)
C9—H9A···O2^ii^	0.99	2.54	3.4055 (16)	145

Symmetry codes: (i) −*x*−1, *y*−1/2, −*z*; (ii) −*x*−1, *y*+1/2, −*z*; (iii) −*x*+1, *y*−1/2, −*z*+1; (iv) −*x*, *y*+1/2, −*z*.
